# Chemical composition, in vitro antioxidant and anti-inflammatory properties of essential oils of four dietary and medicinal plants from Cameroon

**DOI:** 10.1186/s12906-016-1096-y

**Published:** 2016-04-07

**Authors:** Florentine Marie-Chantal Ndoye Foe, Tatiana Flore Kemegni Tchinang, Ascencion Maximilienne Nyegue, Jean-Pierre Abdou, Abel Joel Gbaweng Yaya, Alembert Tiabou Tchinda, Jean-Louis Oyono Essame, François-Xavier Etoa

**Affiliations:** 1 Department of Biochemistry, Faculty of Science, University of Yaounde I, P.O. Box 812, Yaounde, Cameroon; 2 Department of Microbiology, Faculty of Science, University of Yaounde I, P.O. Box 812, Yaounde, Cameroon; 3 Institute of Biomolecules Max Mousseron, “Equipe Glyco et nanovecteurs pour le ciblage thérapeutique”, Faculty of Pharmacy, University of Montpellier II, Montpellier, France; 4 Institute of Medical Research and Medicinal Plants Studies, P.O. Box 8013, Yaounde, Cameroon; 5 Department of Organic Chemistry, Faculty of Science, University of Yaounde I, P.O. Box 812, Yaounde, Cameroon

**Keywords:** Essential oils, Chemical composition, Sulphur-containing plants, In vitro antioxidant, Anti-inflammatory activity

## Abstract

**Background:**

In the Cameroonian traditional medicine, plants of the Capparidaceae, Euphorbiaceae and Liliaceae families are used to treat several metabolic diseases. These plants are rich in various compounds belonging to the glucosinolates and thiosulfinates family. Till date, very little studies have been done aiming at assessing the antioxidant and inflammatory properties of the essential oils (EOs) of these plants. Essential oils are volatile extracts produced by secondary metabolism. They are usually constituted of terpens and may also contain specific non terpenic components such as glucosinolates and thiosulfinates for the species that are being considered in the present study. This study highlights and compares the chemical composition, antioxidant and anti-inflammatory properties of the essential oils of the stem barks of Drypetes gossweileri (Euphorbiaceae), roots of Pentadiplandra brazzeana (Capparidaceae), red bulbs of Allium cepa and Alium sativum (Liliaceae) collected in Cameroon (Central Africa).

**Methods:**

The essential oils were extracted by hydrodistillation and analyzed by gas chromatography (GC) and gas chromatography coupled to mass spectrometry (GC-MS). In vitro antioxidant activities were determined using the radical scavenging assay, total phenolic content, ferric reducing antioxidant power (FRAP) assay and determination of antioxidant activity index (AAI) according to the method described by Scherer and Godoy. The anti-inflammatory activities were evaluated using albumin denaturation method. Differences (*p* < 0.05) between the experimental and the control groups were evaluated using one way analysis of variance (ANOVA) followed by Tukey’s test for multiple comparisons.

**Results:**

The main components of *Allium sativum* essential oil were diallyl trisulfide (41.62 %), diallyl disulfide (19.74 %), allyl methyl trisulfide (12.95 %), diallyl sulfide (7.1 %) and diallyl tetrasulfide (4.22 %). Those of *Allium cepa* essential oil were diallyl trisulfide (22.17 %), dipropyl trisulfide (11.11 %), 2-methyl-3,4-dithiaheptane (9.88 %), methyl propyl trisulfide (8.14 %), dipropyl tetrasulfide (8.07 %) and 2-propenyl propyl disulfide (5.15 %). *Drypetes gossweileri* and *Pentadiplandra brazzeana* essential oils presented similar chemical compositions as compared with benzylisothiocyanate content (63.19 and 97.63 % respectively), but differed in benzylcyanide content (35.72 and 0.86 % respectively). The essential oils were rich in phenolic compounds in the following order *Allium sativum* < *Allium cepa < D. gossweileiri < P. brazzeana*. The essential oils exhibited high antioxidant and DPPH radical scavenging effect but low ferric reducing power activity. Moreover, the four essential oils showed anti-inflammatory activities (by heat denaturation of Bovine Serum Albumin). The anti-inflammatory activities of *P. brazzeana* and *A. cepa* essential oils were comparable but higher than those of *D. gossweileri* and sodium diclofenac used as a reference non-steroidal anti-inflammatory drug.

**Conclusion:**

The essential oils of the plants were rich in organosulfur compounds. These compounds were probably responsible for their appreciable antioxidant and anti-inflammatory activities. Due to their antioxidant and anti-inflammatory properties, the essential oils of some of these plants might be used as natural additives in the pharmaceutical, cosmetic and agro-industries.

## Background

*Allium* species, especially *Allium* vegetables are characterized by their high content in thiosulfinates (TNs). The TNs or alkane (ene) thial-*S*-oxide are formed by the action of the enzyme alliinase (E.C.4.4.1.4) from their respective S-alk (en) yl cysteine sulfoxides. However, depending on the *Allium* species and the environmental conditions, the TNs formed are degraded to various polysulfides including diallyl, methyl allyl, and diethyl mono-, di-, tri-, tetra-, penta-, and hexasulfides, vinyldithiins, (*E*)- and (*Z*)-ajoene [1]. The organosulfur compounds in *Allium* are water-soluble (S-allylcysteine and S-allylmercaptocysteine) and lipid-soluble compounds (alliin, diallyl sulfide, triallyl sulfide, diallyl disulfide, diallyl polysulfides) [2, 3]. TNs exhibit different degrees of antimicrobial activity and are found to be effective antioxidants in terms of scavenging [1].

In damaged plant cells, notably those of Capparidaceae and Euphorbiaceae families, glucosinolates (GLs) are transformed by the endogenous myrosinase (EC 3.2.1.147) to produce a number of compounds depending on the precursor glucosinolate and the environmental conditions [4]. GLs form a variety of hydrolysis products including isothiocyanates, oxazolidine-2-thiones, nitriles, epithionitriles, and thiocyanates [5]. Certain GLs (sulforaphane and other isothiocyanates) are well known for their diverse biological activities ranging from bactericidal, nematocidal, fungicidal, insecticidal, antioxidant, antimutagenic, antiproliferative and allelophatic properties [6, 7].

Oxidation is a chemical reaction involving the transfer of an electron from electron rich to electron deficient entity. The electron deficient molecule is termed an oxidizing agent [8]. An antioxidant is a substance capable of preventing or slowing the oxidation of other molecules. Substances which protect biomolecules from free radical-mediated damage both in vitro and in vivo fall under this category. Reactive oxygen species (ROS) and reactive nitrogen species (RNS) are generated during irradiation by UV light, X and γ rays, products of metal-catalyzed reactions, which are present as pollutants in the atmosphere, produced by neutrophils and macrophages during inflammation process, by-products of mitochondria-catalyzed electron transport reactions and other mechanisms [9]. ROS at higher concentration (termed oxidative stress) are important mediators of cell structure damages including lipids, membranes, proteins and nucleic acids [10]. The harmful effects of ROS are balanced by the antioxidant action of non-enzymatic antioxidants in addition to antioxidant enzymes [11]. Thus, ROS are important mediators that provoke or sustain inflammatory process and consequently, their neutralization by antioxidants and free radical scavengers can attenuate inflammation [12, 13]. Both inflammation and free radical damage are inter-related aspects that influence each other.

Inflammation is a protective and defense mechanism of the body to a physical, chemical or biological agression. It is important when it does not last for long. Its main purpose is to destroy the injurious agent and/or minimize its harmful effect by limiting its propagation [14]. Inflammatory response occurs in two distinct phases: an acute and a chronic. The acute phase is characterized by local vasodilatation, increased capillary permeability and release of inflammatory mediators like histamine, serotonin and prostaglandins. The Chronic phase is characterized by infiltration of leukocytes and phagocytic cells [15]. This results in tissue degeneration and fibrosis. In the market, most of the available conventional drugs to relieve oxidative stress and to treat inflammatory-related diseases are either steroidal or non-steroidal anti-inflammatory chemicals. Though these drugs are potent enough, long-term administration is required for treatment of chronic diseases. Furthermore, these drugs might lead to severe side effects such as gastric intolerance, bone marrow depression, water and salt retention due to prolonged use [16]. It is in this light that natural remedies with very little side effects, proven efficacy and safety are sought for as substitutes for chemical therapies. Therefore, sulfur-containing plants selected for this study were chosen by virtue of their use in traditional medicine in West Cameroon in the treatment of several inflammatory skin disorders and inflammatory-related diseases.

*Allium sativum* L. and *Allium cepa* L. (Liliaceae) commonly known as garlic and onion respectively are bulbous herbs used as food item, spice and medicine in different parts of the world.*Allium sativum* can also be used in homemade cosmetics. It has been employed for its diverse biological activities including anti-carcinogenic, antiatherosclerotic, antithrombotic, antimicrobial, anti-inflammatory and antioxidant effects [17–21]. The major beneficial effects of *Alliums* are ascribed to the high content in organosulfur compounds produced when *Allium sativum* or *Allium cepa* tissue is damaged and the odorless precursors are converted by the alliinase enzyme and lachrymatory-factor synthase [22, 23]. The most important sulfur-containing compound in *Allium cepa* bulbs is the amino acid cysteine and its derivatives. *A. cepa* also contains high levels of flavonoids mainly quercetin as well as other phenolic compounds [24]. In contrast to *Allium cepa*, *Allium sativum* mainly contains allicin derivatives such as ajoene, vinyldithiins, and alkenyl-sulphides such as diallyl disulfide and diallyl trisulfide depending on the type of solvent used for the extraction [22, 25].

*Drypetes gossweileri* is a dioic plant of the Euphorbiaceae family with an average size of about 30 to 40 m. The color of the stem bark turns from grey-green to yellowish-green and it gives a strong characteristic odor when cut [26]. It has been used in traditional medicine for treating fever, malaria, intestinal worms in Central Africa [27, 28]. Phytochemical studies conducted by Ngouana et al. [29] revealed the presence of metabolites such as alkaloids, phenols, flavonoids, saponines, anthraquinones, tannins, anthocyanins, sterols, lipids and essential oils. Phytochemical analysis of the extracts from previous studies indicated the presence of steroids, triterpenoids, alkaloids, saponins with antimicrobicidal properties [30]. Many research works describe the antibacterial [31, 32], antifungal [29, 33], antioxidant and antiradical [32, 34] effects of *D. gossweileri*. It is is also used in pest management [35].

*Pentadiplandra brazzeana* Baill (Capparidaceae) is a climber found in Western and Central Africa [36], the berries of which are eaten and used as a sweetener of beverages [37]. Root, seed, and leaf extracts of *P. brazzeana* are known to contain benzyl-, 3-methoxybenzyl-, 4methoxybenzyl-, 3,4-dimethoxybenzyl-, and indole-type glucosinolates [38]; and the essential oil obtained from its roots is mainly constituted of benzylisothiocyanate and benzylcyanide [32, 39–41]. Its root essential oil (EO) has been proven to possess free-radical scavenging [32, 40, 42], antibacterial [32, 42], antifungal [41, 42] and anti-inflammatory activities [42]. In previous studies, the aqueous extract of *P. brazzeana* root was shown to possess androgenic activity [43]. Moreover, the crude extract of *P. brazzeana* root as well as the compounds isolated from it (thiourea and urea) exhibited a moderately strong antiplasmodial activity against two *Plasmodium falciparum* strains [44].

The antioxidant and anti-inflammatory activities of some *Allium* species grown in other countries have been analyzed, but no information is available on the in vitro antioxidant activities of red bulb *A. cepa* and red bulb *A. sativum* species growing in Cameroon. Antioxidant activities have already been studied using the DPPH (2, 2-diphenyl-1-picrylhydrazyl) scavenging method and the *β*-carotene bleaching test [40, 42], but a different method is used here to confirm it. In addition, anti-inflammatory activity has not yet been reported for *D. gossweileri* stem barks. Moreover, considering that antioxidants and free radical scavengers can also exert an anti-inflammatory effect [13], the essential oil of *D. gossweileri* stem barks were also assessed for these activities in comparison with that obtained from *A. cepa* and *A. sativum*, two plants well-known for their antioxidant and anti-inflammatory activities. Therefore, the purpose of this study was to determine the chemical composition, antioxidant and anti-inflammatory potential of the aforementioned sulfur-containing plants in order to confirm their efficacy in the treatment of inflammatory disorders as claimed in Cameroonian ethnomedicine.

## Methods

### Chemical reagents and solvents

Folin-Ciocalteu reagent, 2, 2-diphenyl-1-picrylhydrazyl (DPPH) and aluminum chloride were purchased from Sigma-Aldrich (Germany). Ascorbic acid and bovine serum albumin were purchased from Sigma-Aldrich (China). All other reagents and solvents were of analytical grade.

### Plant materials and extraction procedure

*A. sativum* red bulbs, *A. cepa* red bulbs, *D. gossweileri* stem barks and *P. brazzeana* roots were purchased in Yaounde (Mokolo and Mfoundi markets) in August 2013. *A. sativum* red bulbs and *A. cepa* red bulbs were harvested at Garoua (North Region of Cameroon). *D. gossweileri* stem barks and *P. brazzeana* roots were harvested at Hawae and Ngomedzap (Center Region of Cameroon) respectively by the vendors. Identification was done at the National Herbarium of Cameroon by comparison with voucher specimens 25742/SRF/Cam, 44810/HNC, 25749/SRF/Cam and 42918/SRF/Cam for *A. cepa*, *A. sativum*, *D. gossweileri* and *P. brazzeana* respectively.

EOs were extracted by hydrodistillation using a Clevenger-type apparatus for 5 h, dried over anhydrous sodium sulfate and then stored at 4 °C until bioassay. The extraction yields were calculated as the ratio of the mass of EO to the mass of the starting plant material and expressed as a percentage (w/w).

### Chemical analysis of the essential oils

The EOs were analyzed by gas chromatography and gas chromatography coupled to mass spectrometry as described by Agnaniet et al. [45].

#### Gas chromatography

GC analysis was performed on a Varian gas chromatograph, model CP-3380, with flame ionization detector containing two silica capillary columns: HP5 J&W Agilent (5 %-Phenyl-methylpolysiloxane) capillary column (30 m × 0.25 mm i.d. × 0.25 μm film) and Supelcowax 10 (polyethylene glycol) fused capillary column (30 m × 0.25 mm i.d. × 0.25 μm film); N_2_ was the carrier gas at 0.8 mL/min; injection type 0.1 μL of pure sample, split ratio 1:100; injector temperature 220 °C, detector temperature 250 °C; temperature program 50-200 °C at 5 °C/min, then kept at 200 °C for 10 min. The linear retention indices of the components were determined relative to the retention times of a series of n-alkanes. The entire set up was coordinated by COMPASS software system that ensured its functioning and follow-up of the chromatographic analysis.

#### Gas chromatography-mass spectrometry

GC-MS analyses were performed using a Hewlett-Packard GC 5890 series II equipped with a HP5 (5 %-Phenylmethylpolysiloxane) fused silica column (30 m × 0.25 mm; film thickness 0.25 μm) and a DB-Wax fused silica column (30 m × 0.25 mm; film thickness 0.25 μm) interfaced with a quadrupole detector (Model 5972); temperature program (50–200 °C at 5 °C/min); injector temperature, 220 °C; MS transfer line temperature, 180 °C; carrier gas, helium at a flow rate of 0.6 mL/min; injection type, split, 1:10 (1 μL 10:100 CH_2_Cl_2_ solution); ionization voltage, 70 eV; electron multiplier 1460 eV; scan range, 35–300 amu; scan rate, 2.96 scan/s.

#### Qualitative analysis

The identification of the constituents was based on comparison of their relative retention times with either those of authentic samples or with published data in the literature [46] and matching their mass spectra with those obtained from authentic samples and/or the NBS75K and Wiley 7th NIST 98 EPA/NIH libraries spectra and literature data [46].

#### Quantitative analysis

The percentage composition of the essential oils was computed by the normalization method from the GC-FID peak areas, assuming an identical mass response factor for all compounds.

### Determination of radical scavenging and antioxidant activity

In order to determine the radical scavenging and antioxidant activities of the EOs, the following assays were applied: total phenolic content determination, FRAP assay and DPPH assay.

#### Determination of total phenolic content (TPC)

The phenolic content in EOs was determined according to the method described by Singleton et al. [47] with slightly modifications. In effect, 10 μg/mL of EOs were used in the analysis. The reaction mixtures were prepared by mixing 60 μL of EO, 2 mL of 10 % Folin-Ciocalteu dissolved in water. For the blank, 60 μL of methanol, 2 mL of 10 % Folin-Ciocalteu reagent were mixed in water. After 30 min of incubation at room temperature, the absorbance was measured at 750 nm using a V-1100 spectrophotometer. Ascorbic acid was used as standard. Experiments were carried out in triplicate. The total phenolic content was expressed in μg ascorbic acid equivalent per mg of EO (μg AAE/mg) using the following equation based on the calibration curve:

$$ \mathrm{Absorbance} = 0.02\ \mathrm{x}\ \mathrm{ascorbic}\ \mathrm{acid}\ \left(\mu \mathrm{g}\right) + 0.04;\ {\mathrm{R}}^2 = 0.99 $$

#### Ferric reducing antioxidant power (FRAP) assay

The reducing power of the EOs was determined in accordance with the method reported by Zhao et al. [48] with slight modifications. 1 mL of each EO was mixed with 2.5 mL of 0.2 M phosphate buffer (pH = 6.6) and 2.5 mL of potassium ferricyanide [K_3_Fe(CN)_6_] (1 %). The mixture was incubated at 50 °C for 20 min. 2.5 mL of 10 % trichloroacetic acid was added and the tubes were centrifuged for 10 min at 3000 rpm. The supernatant (1.25 mL) was mixed with 1.25 mL of distilled water and 0.25 mL of 0.1 % ferric chloride solution. The absorbance was measured at 700 nm in a V-1100 spectrophotometer. Higher absorbance of the reaction mixture indicated greater reducing power. Experiments were carried out in triplicate. The results were expressed in μg ascorbic acid equivalent per mg of EO (μg AAE/mg) using the equation below based on the calibration curve:

$$ \mathrm{Absorbance} = 0.29\mathrm{x}\ \mathrm{ascorbic}\ \mathrm{acid}\ \left(\mu \mathrm{g}\right) + 0.77;\ {\mathrm{R}}^2 = 0.98 $$

#### DPPH Radical scavenging assay (RSA)

The free radical scavenging activity of the EOs was evaluated as described by Sanchez et al. [49] with slight modifications, based on the ability of test compounds to neutralize DPPH radical. The conversion of the stable violet DPPH radical into a yellow reduced form (DPPH/H^+^) is observed simultaneously. The test samples were prepared in methanol and 100 μL of each sample (1.25 to 10 μg/mL for essential oils) was added to 1900 μL of freshly prepared 2, 2-diphenyl-1-picrylhydrazyl (DPPH) solution (50 mg/L) in pure methanol. Ascorbic acid was used as a positive control and 2 mL of 50 mg/L DPPH/methanol solution was used as negative control. The content of each preparation was mixed and incubated at room temperature in a dark cupboard for 10 min. The absorbance was measured at 517 nm in a V-1100 spectrophotometer. All tests were carried out in triplicate. The radical scavenging activity (RSA) was calculated as a percentage of DPPH radical scavenging, using the equation here below:

$$ \%\ \mathrm{R}\mathrm{S}\mathrm{A} = \left[{\mathrm{Absorbance}}_{\mathrm{blank}}\hbox{--} {\mathrm{Absorbance}}_{\mathrm{sample}}\Big)/{\mathrm{Absorbance}}_{\mathrm{blank}}\right]\ \mathrm{x}100. $$

Where A_blank_ is the absorbance of the control (containing all reagents except the test sample) and A_sample_ is the absorbance of tested EO solution.

The SC_50_ values (concentration of sample required to scavenge 50 % of free radicals) were calculated from the regression equations derived by the least-square method and prepared from the different concentrations of both ethanol extracts and essential oils. The higher the SC_50_ the lower the antioxidant activity of the assayed sample. Ascorbic acid was used as reference.

The antioxidant activity was then calculated and expressed as the antioxidant activity index (AAI): AAI = final concentration of DPPH in the control sample (μg.ml^−1^)/SC_50_ (μg.ml^−1^). Scherer and Godoy’s criteria were considered [50] depending on whether the EOs showed weak antioxidant activity (AAI < 0.5), moderate antioxidant activity (AAI, between 0.5 and 1.0), strong antioxidant activity (AAI, between 1.0 and 2.0) and very strong antioxidant activity when AAI > 2.0.

### Determination of the anti-inflammatory activity: inhibition of albumin denaturation

Anti-denaturation assay was conducted as described by Biswakanth et al. [51] with slight modifications. The reaction mixture consisted of EO samples at different concentrations and 1 % bovine serum albumin (BSA) fraction prepared in saline phosphate buffer (pH = 7.4). The pH of the reaction mixture was adjusted to 6.8 using small amounts of glacial acetic acid. The test tubes were incubated at 72 ° C for 5 min and then cooled for 10 min. The absorbance of these solutions was determined using a spectrophotometer (Mapada V-1100) at a wavelength of 660 nm. The experiment was performed in triplicate. The percentage inhibition of precipitation (denaturation of the protein) was determined on a percentage basis relative to the control using the formula:

$$ \mathrm{Percentage}\ \mathrm{of}\ \mathrm{inhibition}\ \mathrm{denaturation} = 100\ \hbox{--}\ \left[\left({\mathrm{Absorbance}}_{\mathrm{sample}}/{\mathrm{Absorbance}}_{\mathrm{control}}\right)\ \mathrm{x}\ 100\right] $$

### Statistical analysis

All the grouped data were statistically evaluated with GraphPad InStat software. Hypothesis testing methods included one way analysis of variance (ANOVA) followed by Tukey’s test for multiple comparisons. *P* values less than 0.05 were considered to be statistically significant. All the results were expressed as mean ± s*.*d. The regression equations and correlation coefficients were obtained by the least-square method.

## Results and discussion

### Extraction yields of essential oils

The extraction yields of the EOs are shown in Table 1. The yield ranged from 0.007 % for *A. cepa* to 0.2 % for *A. sativum* bulbs. The extraction yield of red bulb *A. cepa* was similar to that previously found by Mnayer et al. [52] (0.006 to 0.013 %) while the yield of *A. sativum* bulbs was similar to that obtained by the same author but, higher (0.09 %) than that observed in a previous study by Khadri et al. [53]. However, the yield obtained in this study represents only half of the yield obtained by Lawrence and Lawrence (0.4 %) [54].

**Table 1:** Relative percentages of constituents and extraction yields of EOs from *A. sativum, A. cepa* bulbs, *D. gossweileri* stem barks and *P. brazzeana* roots

Components	RI	Relative percentage (%)			
		*A. cepa*	*A. sativum*	*D. gossweileri*	*P. brazzeana*
2,4-dimethylthiophene	884	-	0.63	0.03	-
Diallyl sulfide	888	1.38	7.10	0.11	-
2,5-dimethyl thiophene	908	0.82	-		-
Allyl methyl disulfide	918	-	-	0.01	-
Methyl propyl disulfide	922	-	1.19	-	-
N,N’-Dimethyl thiourea	951	-	-	0.01	-
Benzaldehyde	962	-	-	0.28	-
dimethyl trisulfide	964	-	0.58	-	-
2-Phenyl furan	978	-	0.48	-	-
Octyl aldehyde	990	2.26	-	-	-
phenylacetaldehyde	1026	2.69	-	0.06	-
Limonene	1029	-	0.62	-	-
2-propenyl propyl disulfide	1032	5.15	0.20	0.02	-
2,5-dimethyl-1,3,4-thiadiazole	1037	-	0.74	-	-
Terpinolene	1059	-	-	0.02	-
Phenylmethanol	1081	-	-	0.01	-
Diallyl disulfide	1088	-	19.74		-
Trans-propenyl propyl disulfide	1091	2.86	-	-	-
Linalool	1101	-	0.88	-	-
Dipropyl disulfide	1109	2.71	0.62	-	-
1-propenyl propyl disulfide	1114	3.77	-	-	-
3,5-dimethyl-1,2,4-trithiolane	1118	1.81	-	-	-
Campholenol	1124	-	-	0.03	0.31
Allyl methyl trisulfide	1149	-	12.95	-	-
Methyl propyl trisulfide	1153	8.14	-	-	-
Benzylcyanide	1157	2.52	-	35.72	0.86
3,4-Dihydro-3-vinyl-1,2-dithiin	1165	-	1.37	-	-
Dimethyl tetrasulfide	1224	1.32	1.59	0.02	-
2,5-Dimethylthiazole	1265	4.62	-	-	-
Diallyl trisulfide	1319	22.17	41.62	-	-
3-Methoxyoctane	1327	-	0.62	-	-
Dipropyl trisulfide	1334	11.11	-	-	-
1-propenyl propyl sulfide	1345	1.26	-	-	-
Allyl propyl sulfide	1377	-	1.30	-	-
Di-1-propenyl sulfide	1382	1.71	2.08	-	-
Benzylisothiocyanate	1393	-	-	63,19	97.63
p-methoxybenzylcyanide	1395	-	0.85	-	1,2
Β-caryophyllene	1398	-	0.23	-	-
2-methyl-3-isothiozolone	1435	2.23	-	--	-
Germacrene D	1484	-	-	0.02	-
γ-cadinene	1506	-	0.39	-	-
2-methyl-3,4-dithiaheptane	1527	9.88	-	-	
Β-sesquiphellandrene	1550	-	-	0.05	-
Diallyl tetrasulfide	1558	-	4.22	-	-
p-methoxybenzylisothiocyanate	1575	3.55	-	-	-
Dipropyl tetrasulfide	1649	8.07	-	-	-
Benzyl sulfide	1856	-	-	0.06	-
Methyl linolenate	2013	-	-	0.06	-
Total identified (%)		98.77	100	99.70	100
EOs extraction yields (%)		0.007	0.2	0.04	0.02

*RI* linear retention indices on a HP5 column; − not found; *EO* essential oils

As regards *D. gossweileri*, its stem bark gave a yield of 0.04 %. This yield was different from those obtained by Eyele et al. (0.2 %) [55]; Agnaniet et al. (0.19 %) [56]; Ngono (0.007 and 0.29 %) and [32] Mbouma (0.023 to 0.088 %) [33].

The roots of *P. brazzeana* gave a yield of 0.02 % which was similar to those found in previous studies by Koudou et al. [39], Ndoye [40], Ngono [32] and Tchinang et al. [41]. However, this yield is lower than those found by Koudou et al. [39] and Nyegue et al. [42], respectively 0.35, 0.2 and 0.13 %.

### Chemical composition

The results of the chemical analysis are presented in Table 1.

GC-MS analysis of *Allium sativum* EO revealed 22 components representing 100 % of the total EO. The major components were diallyl trisulfide (41.62 %), diallyl disulfide (19.74 %), allyl methyl trisulfide (12.95 %), diallyl sulfide (7.1 %) and diallyl tetrasulfide (4.22 %). This chemical profile was closed to that obtained by Mnayer et al. [52], but with slight difference in that diallyl disulfide (37.90 %) was predominant compared to diallyl trisulfide (28.06 %), while the allyl methyl trisulfide (7.26 %) represented almost half of its content in the previous study [52]. Furthermore, studies conducted by Khadri et al. [53] showed that allyl methyl trisulfide (34.61 %) and diallyl disulfide (31.65 %) were rather the major constituents of the EO of *Allium sativum*. However, it is worth noting that, in the two previous studies [52, 53], allyl methyl disulfide was present in the chemical composition of the *Allium sativum* essential oil, contrarily to this study where it is pratically absent. Nonetheless, different studies on the chemical composition of *A. sativum* essential oil showed that diallyl disulfide and diallyl trisulfide are the two major compounds of the EO of *A. sativum* [52, 57, 58].

Twenty one constituents representing 98.77 % of the total EO were identified in the *Allium cepa* EO. The main components were diallyl trisulfide (22.17 %), dipropyl trisulfide (11.11 %), 2-methyl-3, 4-dithiaheptane (9.88 %), methyl propyl trisulfide (8.14 %), dipropyl tetrasulfide (8.07 %) and 2-propenyl propyl disulfide (5.15 %). The chemical composition of *A. cepa* used in this study differed greatly from previous reports in which dipropyl disulfide was reported to be the major compound present [52, 57].

Analysis of the chemical compositions of the EO of *D. gossweileri* stem barks revealed 17 compounds representing 99.70 % of the total EO. Benzylisothiocyanate was the main component, representing 63.19 % followed by benzylcyanide (35.72 %). These results are in agreement with those of Eyele et al. [55], Ngono [32] and Mbouma [33] who documented that benzylisothiocyanate (56.5 to 95.6 %) and benzylcyanide (4.3 to 32.3 %) were also the major compounds of *D. gossweileri* EO. However, Agnaniet et al., [56], reported that benzylcyanide (73.7 %) was the main compound in *D. gossweileri* stem bark EO from Gabon, instead of benzylisothiocyanate (5.2 %).

As for the EO of *P. brazzeana* roots, four components were identified, representing 100 % of the total oil. The main compound found was benzylisothiocyanate (97.63 %). Benzylisothiocyanate has been reported by Ndoye [40], Nyegue et al. [41], Ngono [32] and Tchinang et al. [41] to be the major compound present in *P. brazzeana* EO.

It was observed that the chemical composition of the aforementioned essential oils differs greatly among genus, and also among the same species. This is possibly due to analytical techniques, chemotypes, and differences in cultivars of the plants, culture climate and other growth conditions which might affect the chemical composition [38, 59].

### Determination of radical scavenging and antioxidant activity

#### Determination of total phenolic content (TPC)

The Total Phenolic Contents (TPC) of *Allium sativum*, *Allium cepa*, *D. gossweileiri* and *P. brazzeana* essential oils (EOs) are presented in Table 2.

**Table 2:** Total phenolic, and ferric reducing power contents of essential oils of *A. cepa, A. sativum, D. gossweileiri, P. brazzeana*

	TPC (μg AAE/mg)	FRAP (μg AAE/mg)
	Essential oil	Essential oil
*A. cepa*	429.33 ± 1.31 ^b^	2.75 ± 0.02 ^b^
*A. sativum*	463.38 ± 1.24 ^a^	5.33 ± 0.01 ^a^
*D. gossweileri*	365.38 ± 0.66 ^c^	0.76 ± 0.03 ^c^
*P. brazzeana*	342.20 ± 0.99 ^d^	0.08 ± 0.03^d^

Values followed by the different superscript letter (a, b, c or d) within the same column are significantly different (*p* < 0.05) according to Tukey’s HSD test. *AAE* ascorbic acid equivalent

From our literature review, there is no scientific investigation on the determination of the TPC of the EOs of *D. gossweileri* stem barks and *P. brazzeana* roots. The results indicate that the TPC of the EOs ranged from 342.20 ± 0.99 to 429.33 ± 1.31 μg AAE/mg.

The highest level of phenolics were found in both *A. sativum* and *A. cepa* EOs (463.38 ± 1.24 and 429.33 ± 1.31 μg AAE/mg), while the lowest contents were recorded in the EOs of *D. gossweileri* stem barks and *P. brazzeana* roots (365.38 ± 0.66 μg AAE/mg and 342.20 ± 0.99 μg AAE/mg). These results do not match with those of Abdel-Salam et al. [60] who showed that TPC of red *A. sativum* are lower than those of red *A. cepa*.

Generally, EOs possess significant secondary metabolites from plants, particularly the active lipophilic compounds. This might be due to the fact that phenolic compounds are often extracted in higher amounts by using polar solvents such as water [61]. Phenolic antioxidants are products of secondary metabolism in plants and their antioxidant activity is mainly due to their redox properties and chemical structure, which might play an important role in chelating transition metals and scavenging free radicals [62]. Consequently, the antioxidant activities of plant extracts are often explained by their total phenolic and flavonoid contents. Also, the sterical structures of antioxidants or free radicals are known to play a more important role in their abilities to scavenge different types of free radicals [63].

#### Ferric reducing antioxidant power (FRAP)

The ferric reducing power in the EOs of *A. cepa, A. sativum, D. gossweileiri, P. brazzeana* is presented in Table 2. Form our literature review; there is no scientific report on the determination of the ferric reducing power of *D. gossweileri* and *P. brazzeana* EOs.

Their ferric reducing capacity ranged from 0.08 ± 0.03 to 2.75 ± 0.02 μg AAE/mg. The highest reducing power was found in *A. sativum* EO (5.33 ± 0.01 μg AAE/mg), followed by the *A. cepa* EO (2.75 ± 0.02 μg AAE/mg), *D. gossweileiri* EO (0.76 ± 0.03 μg AAE/mg) and finally, EO (0.08 ± 0.03 μg AAE/mg) of *P. brazzeana*. These results are comparable to that of Benkeblia et al. [64] who found that *A. cepa* and *A. sativum* showed the highest reducing capacity with 107 and 196 %, respectively. It is worth mentioning that the ferric reducing antioxidant power of *D. gossweileiri* EO was 10 folds greater than that of *P. brazzeana* EO. This might be ascribed to the presence of more phenolic compounds [65] in *D. gossweileiri* than in *P. brazzeana*. The ability to reduce the ferricyanide complex of Fe^3+^ to the ferrous (Fe^2+^) form might be attributed to the hydrogen donating ability of phenolic compounds [66], which is indicative of the presence of a reducing agent [67]. In addition, the number and position of the hydroxyl groups in phenolic compounds also govern their antioxidant activity [68]. However, Amagase et al. [69] argued that this antioxidant activity could be attributed to the organosulfur compounds.

#### DPPH Radical scavenging assay (RSA)

Tables 3 and 4 show the radical scavenging activity (RSA) of the EOs of *A. cepa, A. sativum, D. gossweileiri* and *P. brazzeana*.

**Table 3:** DPPH radical scavenging activity and AAI of *A. cepa, A. sativum, D. gossweileri, P. brazzeana* essential oils and ascorbic acid

	Regression curve’s equations	Coefficient of determination : R^2^	SC_50_(μg/ml)	AAI
*A. cepa*	y = 192.3 × + 11.96	0.927	0.20	12.626
*A. sativum*	y = 191.6 × + 12.73	0.919	0.19	12.886
*D. gossweileri*	y = 194.0 × + 11.10	0.931	0.20	12.821
*P. brazzeana*	y = 190.2 × + 14.15	0.904	0.19	13.298
Ascorbic acid	y = 19.12 × + 12.19	0.934	1.98	1.262

Linear regression analysis was used to calculate SC_50_ value

*SC*_*50*_ Scavenging concentration (μg/mL) at 50 %, *AAI* antioxidant activity index

**Table 4:** Relationship between TPC and FRAP, TPC and RSA

	Relationship between TPC and FRAP, FRAP = f (TPC)		Relationship between TPC and RSA, SC_50_ = f (1/TPC)	
	Regression curve Equation	Coefficient of determination : R^2^	Regression curve equation	Coefficient of determination : R^2^
Essential oil	y = 0.016× - 0.434	0.22	y = 0.782 × + 0.189	0.21

The stable free radical DPPH was used to test the ability of the EOs and ascorbic acid to donate hydrogen ion. The scavenging effects of the EOs on DPPH radical increased as the EO concentration increased. All the EOs were able to reduce the stable free radical 2, 2-diphenyl-1-picrylhydrazyl (DPPH) to the yellow diphenylpicrylhydrazine with varying degrees of scavenging capacities. Great bleaching action (from purple to yellow) reflected a higher antioxidant activity and thus a lower SC_50_. The SC_50_ values increased in the following order: *P. brazzeana* < *A. sativum* < *D. gossweileri* < *A. cepa* < ascorbic acid. These results indicate that EOs exhibited a significant DPPH radical scavenging activity about 10 folds more active than ascorbic acid. Thus, the EOs were found to be better antioxidants. These results are in agreement with the findings of Agnaniet et al. [56], Ngono [32] and Mnayer et al. [52], but in disaccordance with those found by Ndoye [40], Nyegue et al. [42], Lee et al. [24] and Abdel-Salam et al. [60]. Mnayer et al. [52] showed that *Allium sativum* EO was a more effective DPPH radical scavenger (SC_50_ = 7.67 mg/mL) than *Allium cepa* EO (SC_50_ = 20.19 mg/mL). On the contrary, Lee et al. [24] and Abdel-Salam et al. [60] reported that *Allium cepa* EO exhibited higher DPPH radical scavenging activity than *A. sativum* EO. Moreover, Negue et al. [42] found that *P. brazzeana* EO exhibited a weak DPPH radical scavenging activity (RSA = 14 % for 1 g/L, SC_50_ = 1.5 mg/mL and SC_50_ = 3.23 mg/mL) in comparison with the results of the present study. However, Ngono [32] stated that although the RSA of both the EO of *P. brazzeana* roots and *D. gossweileri* stem barks were weak, the RSA of *P. brazzeana* EO was higher than that of *D. gossweileri* EO. The major components of the EO (benzylisothiocyanate and benzylcyanide) of these two plants could not be responsible for the antiradical activity, but it might be due to the synergetic action between different components or the contribution of some minor components [41]. It has been shown that benzylisothiocyanate and benzylcyanide tested individually were found to be less active than the whole EO.

Using Scherer and Godoy’s criteria [49], EO showed very strong antioxidant activity (AAI > 2), while ascorbic acid showed a strong antioxidant activity index (1 < AAI < 2).

It is interesting to note that there were weak linear correlations between the total phenolic concentration and the FRAP values or DPPH radical scavenging percentage, with coefficients, R^2^ = 0.22 and 0.21 respectively (Table 4). These results show that most antioxidant compounds might be organosulfur compounds. Indeed, the antioxidant activity of *Allium* species is attributed partly to its sulfur compounds, which represent the main constituents of these EO [57, 70, 71]. Amagase et al. [69] reported that diallyl polysulphides contributed to the antioxidant properties of the EOs. In addition, the antioxidant activity is also related to other minor bioactive compounds: dietary fibers, microelements (especially selenium) and polyphenols [72] which potentialize antioxidant activity. The different antioxidant activities between these EO might be due to the variability of the chemical composition and sulfides concentration. It might also be attributed to the presence and synergitic action of the different trace compounds.

### Determination of the anti-inflammatory activity

Anti-denaturation of bovine serum albumin method was used to evaluate the anti inflammatory property of the EOs of *A. cepa, A. sativum, D. gossweileiri* and *P. brazzeana*. The results are summarized in Table 5. All the EOs protected the BSA against heat induced denaturation. The percentage of BSA protection against heat increased with increasing concentration. The present findings show a concentration dependent inhibition of protein (albumin) denaturation by EOs from 3.125 to 125 μg/mL. Sodium diclofenac at the same concentration range was used as the reference drug and it also exhibited a concentration dependent inhibition of protein denaturation. Indeed, the effect of sodium diclofenac against heat denaturation of BSA was found to be about 2 folds lower than that of *A. cepa* and *P. brazzeana* EO. *P. brazzeana* EO was found to be 2 folds more active than *D. gossweileri* EO. The anti-inflammatory activity of *P. brazzeana* EO was to a lesser extent comparable to that of *A. cepa*. These observations were confirmed by comparing their IC_50_ values.

**Table 5:** Effect of essential oil on BSA denaturation inhibitory activity, percentage inhibition compared to sodium diclofenac

	*A. cepa*	*A. sativum*	*D. gossweileri*	*P. brazzeana*	Diclofenac sodium
Concentration (μg/ml)	Inhibition percentage (%)				
3.125	7.33 ± 1.15^b^	4.00 ± 0.00^a^	4.00 ± 0.00^a^	7.56 ± 2.00^b^	7.33 ± 0.00^b^
6.25	9.33 ± 1.15^a^	6.00 ± 2.00^a^	8.67 ± 2.31^a^	16.477 ± 1.15^b^	9.33 ± 1.15^a^
12.5	14.00 ± 2.00^a^	14.00 ± 2.00^a^	13.33 ± 3.05^a^	26.94 ± 2.00^b^	14.00 ± 0.00^a^
25	33.33 ± 1.15^c^	22.66 ± 2.30^b^	19.33 ± 1.15^a^	38.95 ± 1.15^d^	18.00 ± 0.00^b^
50	54.67 ± 3.05^c^	38.00 ± 2.00^b^	28.00 ± 2.00^a^	52.52 ± 1.15^c^	24.00 ± 0.00^a^
75	82 ± 1.04^e^	57 ± 1.08^c^	42 ± 0.11^b^	72.87 ± 0.98^d^	36 ± 2.00^a^
100	-	76 ± 0.91^c^	56 ± 0.76^b^	-	48 ± 1.00^a^
125	-	95 ± 1.02^c^	70 ± 2.14^b^	-	60 ± 1.73^a^
Regression curve equations	y = 1.070x + 2.579	y = 0.753x – 3.637	y = 0.525x + 3.642	y = 0.907x + 8.587	y = 0.430x + 5.090
	R^2^ = 0.991	R^2^ = 0.976	R^2^ = 0.991	R^2^ = 0.973	R^2^ = 0.982
IC_50_ value (μg/mL)	44.31	61.577	88.30	45.66	104.44

Linear regression analysis was used to calculate IC_50_ value

Values followed by the same superscript letter (a, b, c, d or e) within the same line are not significantly different (*p* > 0.05) according to Tukey’s HSD test

To the best of our knowledge, no study on the in vitro anti-inflammatory properties of the EOs of *D. gossweileri* has been published. However, [42] reported that the anti-inflammatory activity of the EO of *P. brazzeana* root is about 100 folds less active (35 ± 5 μg/mL) than that of nordihydroguaiaretic acid (0.23 ± 0.02 μg/mL) against the soybean 5-lipoxygenase enzyme. The IC_50_ of *P. brazzeana* EO found in this study is slightly higher (45.66 μg/mL) than that reported by Nyegue et al. [42] using a different method. This means that the EO sample used by Nyegue et al. [42] was slightly more active than the present EO sample. This could be explained by the fact that the EO sample used by the aforementioned author contained more compounds (11) than the EO sample used in this investigation, which contained only 4 components (Table 1). Concerning the antiradical, anti-inflammatory activities of the EO of *P. brazzeana* and *D. gossweileri*, these activities might be due to the synergetic action of several components or the contribution of trace components. Nyegue et al. [42] reported that the two main components (benzyisothiocyanate and benzylcyanide) tested individually were found to be less active than the whole essential oil.

## Conclusion

In order to valorize some organosulfur plants from Cameroon as dietary, cosmetic and pharmaceutical additives, the chemical composition, in vitro antioxidant and anti-inflammatory activities of the essential oils of *A. sativum, A. cepa, D. gossweileri* and *P.brazzeana* were investigated in this study. This paper reveals that the essential oils of *D. gossweileri* stem barks and *P. brazzeana* roots as well as those of the red bulbs of *A.sativum* and *A. cepa* are all effective antioxidant and anti-inflammatory compounds. The findings of this study support the popular use of these plants against inflammatory skin disorders and inflammatory-related diseases in West-Cameroon, though additional studies are necessary to test other enzymatic systems that trigger the development of the inflammatory process and also address their mechanism of action.

### Availability of data and materials

The datasets supporting the conclusions of this article are presented in this main paper. Plant materials used in this study have been identified at the Cameroon National Herbarium where voucher specimens are deposited.

### Consent for publication

Not applicable in this section.

### Ethic approval and consent to participate

Not applicable in this section.

## Abbreviations

%: percentage
*A. cepa*: *Allium cepa*
*A. sativum*: *Allium sativum*
AAE: ascorbic acid equivalent
AAI: antioxidant activity index
BSA: bovine serum albumin
*D. gossweileri*: *Drypetes gossweileri*
DPPH: 2, 2-diphenyl-1-picrylhydrazyl
EO: essential oils
eV: electron volt
FRAP: ferric reducing antioxidant power
GC: gas chromatography
GC-FID: gas chromatography-flame ionization detector
GC-MS: gas chromatography-mass spectrometry
GLs: glucosinolates
LRI: linear retention indices found on a hp5 column
mg: milligramme
mL: milliliter
*P. brazzeana*: *Pentadiplandra brazzeana*
pH: potential of hydrogen
R^2^: correlation cefficient
RSA: radical scavenging activity
SC_50_: scavenging concentration at 50 %
TNs: thiosulfinates
TPC: total phenolic content
w/w: weigh: weight
μg: microgramme
μL: microliter

## References

[1] Benkeblia N, Lanzotti V (2007). *Allium* thiosulfinates: chemistry, biological properties and their potential utilization in food preservation. Food.

[2] Lawson LD, Wang ZJ, Hughes BG (1991). Identification and HPLC quantitation of the sulfides and dialk (en)yl thiosulfinates in commercial garlic products. Planta Med.

[3] Matsuura H, Lachance PA (1997). Phytochemistry of garlic horticultural and processing procedures. Neutraceuticals: designer foods III. Garlic, Soy and licorice.

[4] Bellostas N, Sorensen AD, Sorensen JC, Sorensen H, Gupta S (2007). Genetic variation and metabolism of glucosinolates in cruciferous oil seed crops. Rapeseed breeding: advances in botanical research.

[5] Halkier BA, Gershenzon J (2006). Biology and biochemistry of glucosinolates. Annu Rev Plant Biol.

[6] Fahey JW, Haristoy X, Dolan PM, Kensler TW, Scholtus I (2002). Sulforaphane inhibits extracellular, intracellular, and antibiotic-resistant strains of *Helicobacter pylori* and prevents benzo[*a*]pyrene-induced stomach tumors. Proc Natl Acad Sci U S A.

[7] Kim JK, Chu MS, Kim SJ, Lee SY, Lim SH, Kweon SJ, Cho HS (2010). Variation of Glucosinolates in vegetable crops of *Brassica rapa* L ssp Pekinensis. Food Chem.

[8] Swaran JSF (2009). Structural, chemical and biological aspects of antioxidants for strategies against metal and metalloid exposure. Oxid Med Cell Longev.

[9] Cadenas E (1989). Biochemistry of oxygen toxicity. Annu Rev Biochem.

[10] Poli G, Leonarduzzi F, Biasi E, Chiarpotto E (2004). Oxidative stress and cell signaling. Curr Med Chem.

[11] Halliwell B (1996). Antioxidants in human health and disease. Annu Rev Nutr.

[12] Delaporte RH, Sanchez GM, Cuellar AC, Giuliani A, Palazzo de Mello JC (2002). Anti-inflammatory activity and lipid peroxidation inhibition of iridoid lamiide isolated from *Bouchea fluminensis* (Vell) Mold (Verbenaceae). J Ethnopharmacol.

[13] Geronikaki AA, Gavalas AM (2006). Antioxidants and anti-inflammatory diseases: synthetic and natural antioxidants with anti-inflammatory activity. Comb Chem High T Scr.

[14] Harsh M (2005). Inflammation and healing.

[15] Hochberg MC, Silman AJ, Smolen JS, Weinblatt ME, Weisman MH (2008). Rheumatology.

[16] Gooch K, Culleton BF, Manns BJ, Zhang J, Alfonso H, Tonelli M (2007). NSAID use and progression of chronic kidney disease. Am J Med.

[17] Augusti KT (1996). Therapeutic values of onion (*Allium cepa* L) and garlic (*Allium sativum* L.). Indian J Exp Biol.

[18] Wargovich MJ, Uda N, Woods C, Velasco M, McKee K (1996). Allium vegetables: Their role in the prevention of cancer. Biochem Soc Trans.

[19] Brace LD (2002). Cardiovascular benefits of garlic (*Allium sativum* L.). J Cardiovasc Nurs.

[20] Hunter R, Caira M, Stellenboom N (2005). Thiolsulfinate allicin from garlic: Inspiration for a new antimicrobial agent. Ann NY Acad Sci.

[21] Leelarungrayub N, Rattanapanone V, Chanarat N, Gebicki JM (2006). Quantitative evaluation of the antioxidant properties of garlic and shallot preparations. Nutr.

[22] Keusgen M, Rabinowitch HD, Currah L (2002). Health and alliums. Allium crop science-recent advances.

[23] Vazquez-Prieto MA, Miatello RM (2010). Organosulfur compounds and cardiovascular disease. Mol Aspects Med.

[24] Lee J, Mitchell AE (2011). Quercetin and isorhamnetin glycosides in onion (*Allium cepa* L.): varietal comparison, physical distribution, coproduct evaluation, and long-term storage stability. J Agric Food Chem.

[25] Rose P, Whiteman M, Moore PK, Yi ZZ (2005). Bioactive S-alk (en) yl cysteine sulfoxide metabolites in the genus Allium: the chemistry of potential therapeutic agents. Nat Prod Rep.

[26] Walker AR, Sillans R, Trochain JL (1961). Les plantes utiles du Gabon.

[27] Ngoupayou J (2003). Contribution to the phytochemical study of two medicinal plants of Cameroon: *Drypetes gossweileri* (Euphorbiaceae) and *Parkia filicoidea* (Mimosaceae), 3rd Cycle Doctoral Dissertation.

[28] Tchinda TA, Sob VST, Schmelzer GH, Schmelzer GH, Gurib-Fakim A (2008). *Drypetes gossweileri* S Moore. Medicinal plants.

[29] Ngouana V, Fokou TPV, Saha FBU, Ngouela SA, Boyom FF, Amvam ZPH (2011). Antifungal activity and acute toxicity of stem bark extracts of *Drypetes Gossweileri* S. Moore (Euphorbiaceae) from Cameroon. Afr J Tradit Complem Altern Med.

[30] Dupont MP, Liabrés G, Delauche C, Tchissambou L, Gastman JP (1997). Sterolic and triterpenoidic constituents of stem bark of *Drypetes* gossweileri. Planta Med.

[31] Ijah UJJ, Oyebanji FO (2003). Effects of tannins and polyphenols of some medicinal plants on bacterial agent of urinary tract infections. Global J Pure Appl Sci.

[32] Ngono EF (2010). Évaluation des activités antibactérienne et antiradicalaire in vitro des huiles essentielles de *Drypetes gossweileri S Moore* et de *Pentadiplandra brazzeana Baill* Mémoire de Master en Biochimie.

[33] Mbouma GF (2012). Caractérisation, mise en évidence des chémotypes de l’huile essentielle des écorces de *Drypetes gossweileri S Moore* récoltées au Cameroun et évaluation de leurs activités antifongiques Mémoire de Master en Biochimie.

[34] Agnaniet A, Mounzeo H, Menut C, Bessiere JM, Criton M (2003). The essential oils of *Rinorea subintegrifolia* O ktze and *Drypetes gosweileri* S Moore occurring in Gabon. Flavour Flagrance J.

[35] Toumnou AL, Seck D, Lakouetene DPB, Bolevane OSF, Gueye MT, Traoré A, Namkosséréna S, Noba K, Sembène M, Syssa-Magalé JL (2003). Chemical characterization and insecticidal activity of ethyl acetate and dichloromethane extracts of *Drypetes gossweileri* against *Sitophilus zeamais*, *Tribolium castaneum* and *Rhyzopertha dominica*. J Life Sci.

[36] Jiofack T, Ayissi I, Fokunang C, Guedje N, Kemeuze V (2009). Ethnobotany and phytomedicine of the upper Nyong valley forest in Cameroon. Afr J Pharm Pharmacol.

[37] Tsopmo A, Ngnokam D, Ngamga D, Ayafor JF, Sterner O (1999). Urea derivatives from *Pentadiplandra brazzeana*. J Nat Prod.

[38] De Nicola GR, Nyegue M, Montaut S, Iori R, Menut C, Tatibouet A, Rollin P, Ndoye C, Amvam ZPH (2012). Profile and quantification of glucosinolates in *Pentadiplandra brazzeana* Baillon. Phytochem.

[39] Koudou J, Sakanga O, Menut C, Bessiere JM (2001). Constituants volatils de l'huile essentielle de Pentadiplandra Brazzaena Baillon de Centrafrique Pharmacogn. Med Tradit Afr.

[40] Ndoye F (2001). Étude chimique et évaluation des propriétés antiradicalaires et antioxydantes des huiles essentielles d’espèces aromatiques tropicales en provenance d’Est du Cameroun.

[41] Tchinang KTF, Ndoye FCF, Nyegue MA (2013). Caractérisation des huiles de *Pentadiplandra brazzeana* (Capparidaceae) et évaluation de leurs activités antifongiques sur *Trichophyton rubrum*. Biosci Proc.

[42] Nyegue M A, Ndoyé F, Amvam Zollo H, Etoa F-X, Agnaniet H, Menut C (2009) Chemical and biological evaluation of essential oil of *Pentadiplandra brazzeana* (*Baill*) roots from Cameroon. Adv Phytother Res. 2002; 91–107

[43] Kamtchouing P, Mbongue FGY, Dimo T, Jantsa HB. Evaluation of androgenic activity of *Zingiber officinale* and *Pentadiplandra brazzeana* in male rats. Asian J Androl. 2002;299–301. 12508133

[44] Ngamba D (2004). Antimalarial secondary metabolites from some Cameroonian Medicinal Plants.

[45] Agnaniet H, Agrebi A, Bikanga R, Makani T, Lebibi J, Casabianca H, Morere A, Menut C (2011). Essential oils of *plectranthus tenuicaulis* leaves from Gabon, source of (R), (E), 6-7-epoxyocimene an unusual chemical composition within the genus plectranthus. Natural Product Communun.

[46] Adams RP (2012). Identification of essential oils by Gas Chromatography Quadrupole Mas Spectroscopy.

[47] Singleton VL, Orthofer R, Lamuela-Raventos RM (1999). Analysis of total phenols and other oxidation substrates and antioxidants by means of Folin-Ciocalteu reagent. Methods Enzymol.

[48] Zhao H, Fan W, Dong J, Lu J, Chen J, Shan L, Lin Y, Kong W (2008). Evaluation of antioxidant activities and total phenolic contents of typical malting barley varieties. Food Chem.

[49] Sanchez-Moreno C, Larrauri JA, Saura-Calixto F (1999). Free radical scavenging capacityand inhibition of wines, grape juices and related polyphenolic constituents. Food Res Int.

[50] Scherer R, Godoy HT (2009). Antioxidant activity index (AAI) by 2, 2-diphenyl-1picrylhydrazyl methods. Food Chem.

[51] Biswakanth K, Suresh KRB, Indrajit K, Narayan D, Asis B, Upal KM, Pallab KH. Antioxidant and in vitro anti-inflammatory activities of *Mimusops elengi* leaves. Asian Pac J Trop Biomed. 2012;976–980.

[52] Mnayer D, Fabiano-Tixier AS, Petitcolas E, Hamieh T, Nehme N, Ferrant C, Fernandez X, Chemat F (2014). Chemical composition, antibacterial and antioxidant activities of six essentials oils from the *Alliaceae* family. Molecules.

[53] Khadri S, Boutefnouchet N, Dekhil M (2010). Antibacterial activity evaluation of *Allium sativum* essential oil compared to different *Pseudomonas aeruginosa* strains in Eastern Algeria. Sci Study and Res.

[54] Lawrence R, Lawrence K. Antioxidant activity of garlic essential oil (*Allium Sativum*) grown in north Indian plains. Asian Pac J Trop Biomed. 2011;1(3).

[55] Eyele M, Menut C, Bessière JM, Lamaty G, Nzé Ekekang L, Denamganai J (1997). Aromatic plants of tropical Central Africa: Benzylisothiocyanates major constituent of bark essential oil of *drypetes gossweileri* S Moore. J Essent Oil Res.

[56] Agnaniet H, Mounzeo H, Menut C, Bessière JM, Criton M (2003). The essential oil of *Rinorea subintegrifolia* OKtze and *Drypetes gossweileri* S. Moore occuring in Gabon. Flavour Flag J.

[57] Corzo-martinez M, Corzo N, Villamiel M (2007). Biological properties of onions and garlic. Trends Food Sci Tech.

[58] Casella S, Leonardi M, Melai B, Fratini F, Pistelli L (2012). The Role of Diallyl Sulfides and Dipropyl Sulfides in the in vitro Antimicrobial Activity of the Essential Oil of Garlic, Allium sativum L, and Leek, Allium porrum L. Phytother Res.

[59] Schulz H, Krüger H (2002). Rapid SPME-GC analysis of volatile secondary metabolites in various wild species of genus *Allium*. J Herbs Spices Med Plants.

[60] Abdel-Salam AF, Shahenda ME, Jehan BA (2014). Antimicrobial and antioxidant activities of red onion, garlic and leek in sausage. Afr J Microbiol Res.

[61] Sultana B, Anwar F, Przybylski R (2007). Antioxidant activity of phenolics components present in barks of *Azadirachta indica*, *Terminalia arjuna*, *Acacia nilotica* and *Eugenia jambolana* Lam trees. Food Chem.

[62] Mohamed AA, Khalil AA, El-Beltagi HES (2010). Antioxidant and antimicrobial properties of kaff maryam (*Anastatica hierochuntica*) and doum palm (*Hyphaene thebaica*). Grasas Aceites.

[63] Guo Q, Rimbach G, Moini H, Weber S, Packer L (2002). ESR and cell culture studies on free radical-scavenging and antioxidant activities of isoflavonoids. Toxicol.

[64] Benkeblia N (2005). Free-radical scavenging capacity and antioxidant properties of some selected onions (*Allium cepa* L.) and Garlic (*Allium sativum* L.) extracts. Braz Arch Biol Techn.

[65] Griffiths GL, Trueman T, Crowther B, Thomas B, Smith B (2002). Onions, a global benefit to health. Phytother Res.

[66] Shimada K, Fujikawa K, Yahara K, Nakamura T (1992). Antioxidative properties of xanthan on the autoxidation of soyabean oil in cyclodextrin emulsion. J Agric and Food Chem.

[67] Duh PD (1998). Antioxidant activity of Budrock (*Arctium laooa* Linn.): its scavenging effect on free radical and active oxygen. J Am Oil Chem Soc.

[68] Rice-Evans CA, Miller NJ, Bolwell PG, Bramley PM, Pridham JB (1995). The relative antioxidant activities of plant-derived polyphenolic flavonoids. Free Radical Res.

[69] Amagase H, Petesch B, Matsuura H, Kasuga S, Itakura Y (2001). Intake of garlic and its bioactive components. J Nutr.

[70] Lampe J (1999). Health effects of vegetables and fruit: assessing mechanisms of action in human experimental studies. Am J Clin Nutr.

[71] Yin M, Cheng W (2003). Antioxidant and antimicrobial effects of four garlic-derived organosulfur compounds in ground beef. Meat Sci.

[72] Lanzotti V (2006). The analysis of onion and garlic. J Chromatogr A.

